# Differential modulation of immune response and cytokine profiles in the bursae and spleen of chickens infected with very virulent infectious bursal disease virus

**DOI:** 10.1186/s12917-015-0377-x

**Published:** 2015-03-25

**Authors:** Mehdi Rasoli, Swee Keong Yeap, Sheau Wei Tan, Kiarash Roohani, Ye Wen Kristeen-Teo, Noorjahan Banu Alitheen, Yasmin Abd Rahaman, Ideris Aini, Mohd Hair Bejo, Pete Kaiser, Abdul Rahman Omar

**Affiliations:** Institute of Bioscience, Universiti Putra Malaysia, Serdang, 43400 Selangor Malaysia; Faculty of Biotechnology and Biomolecular Sciences, Universiti Putra Malaysia, Serdang, 43400 Selangor Malaysia; Faculty of Veterinary Medicine, Universiti Putra Malaysia, Serdang, 43400 Selangor Malaysia; The Roslin Institute and R(D)SVS, University of Edinburgh, Easter Bush, Midlothian, EH25 9RG UK

**Keywords:** vvIBDV, Viral load, GeXP, Real-time PCR, Pro-inflammatory cytokines, Chemokines

## Abstract

**Background:**

Very virulent infectious bursal disease virus (vvIBDV) induces immunosuppression and inflammation in young birds, which subsequently leads to high mortality. In addition, infectious bursal disease (IBD) is one of the leading causes of vaccine failure on farms. Therefore, understanding the immunopathogenesis of IBDV in both the spleen and the bursae could help effective vaccine development. However, previous studies only profiled the differential expression of a limited number of cytokines, in either the spleen or the bursae of Fabricius of IBDV-infected chickens. Thus, this study aims to evaluate the *in vitro* and *in vivo* immunoregulatory effects of vvIBDV infection on macrophage-like cells, spleen and bursae of Fabricius.

**Results:**

The viral load was increased during the progression of the *in vitro* infection in the HD11 macrophage cell line and *in vivo*, but no significant difference was observed between the spleen and the bursae tissue. vvIBDV infection induced the expression of pro-inflammatory and Th1 cytokines, and chemokines from HD11 cells in a time- and dosage-dependent manner. Furthermore, alterations in the lymphocyte populations, cytokine and chemokine expression, were observed in the vvIBDV-infected spleens and bursae. A drastic rise was detected in numbers of macrophages and pro-inflammatory cytokine expression in the spleen, as early as 2 days post-infection (dpi). On 4 dpi, macrophage and T lymphocyte infiltration, associated with the peak expression of pro-inflammatory cytokines in the bursae tissues of infected chickens were observed. The majority of the significantly regulated pro-inflammatory cytokines and chemokines, in vvIBDV-infected spleens and bursae, were also detected in vvIBDV-infected HD11 cells. This cellular infiltration subsequently resulted in a sharp rise in nitric oxide (NO) and lipid peroxidation levels.

**Conclusion:**

This study suggests that macrophage may play an important role in regulating the early expression of pro-inflammatory cytokines, first in the spleen and then in the bursae, the latter tissue undergoing macrophage infiltration at 4 dpi.

## Background

Infectious bursal disease is an important viral disease, resulting in an acute and contagious infection on poultry farms. The causative agent is infectious bursal disease virus (IBDV) which belongs to the *Birnaviridae* family. IBDV is categorised into different types based on virulence, and the very virulent (vv) strain is the most acute and lethal. Presently, vvIBDV outbreaks have been reported in various countries, causing severe economic losses in the poultry industry. This virus can overcome maternally derived antibodies (MDA) and can cause 80 to 100 percent mortality in susceptible chickens [1]. The main targets of IBDV are IgM-bearing B cells, found in the gut-associated lymphoid organs and the bursae of Fabricius. Following infection, depletion of B cells due to viral-induced apoptosis in B cell occurred, causing severe immunosuppression in young chickens [2]. The susceptibility of T cells to IBDV is not well characterised. However, T cells are a crucial component in the immunopathogenesis of IBDV, as infiltration of CD4^+^ and CD8^+^ cells was detected in the bursae between 1 to 10 dpi [3,4], without affecting the population of these cells in spleen and peripheral blood [5,6]. In addition, infection of one-day-old chicks results in a rapid decline in B cell numbers in the peripheral blood and spleen. Apart from B cells, macrophages can also be infected by IBDV *in vitro* [7].

Classical and vvIBDV induce differential host immune responses [8-10]. However, both virus strains upregulate the expression of Th1-like and pro-inflammatory cytokines, such as IL-12, IFN-γ, IL-1β, IL-6, iNOS, and IL-18, in bursae tissue, but vvIBDV-induced IFN-γ is expressed at greater magnitude, compared to classical IBDV. Modulation of IFN-γ gene expression that leads to subsequent production of nitric oxide by macrophages [11], probably associated with the bursae-infiltrating CD4^+^ and CD8^+^ cells [12]. Furthermore, Tippenhauer *et al.* [13] reported different strains of IBDV differentially regulated levels of types I and II IFN expression in infected spleens and bursae. However, the contribution of macrophages to the cytokines and chemokines induced following IBDV infection, *in vitro* or *in vivo*, is still unknown. In this study, the immunomodulatory effect of vvIBDV on the expression of selected cytokines, chemokines and other immune-related genes was evaluated, and correlated with viral load, in *in vitro* infection of the HD11 macrophage cell line, and *in vivo* infection, specifically in the spleens and bursae, of specific-pathogen-free (SPF) chickens.

## Methods

### Propagation of vvIBDV strain UPM0081 in SPF embryonated chicken eggs

The UPM0081 strain was first isolated during an IBD outbreak in 2000 in Kelantan, a northern state of peninsular Malaysia. Based on the virus pathogenicity and the VP2 sequence analysis (NCBI Acc. No. AY520910), it was characterised as a vvIBDV strain [14]. The virus was propagated using nine-day-old embryonated SPF chicken eggs and stored at -80°C. The median embryo infective dose of the virus (EID_50_) was calculated using the Reed-Muench method [15].

### In vitro *vvIBDV infection of HD11 cells*

The chicken monocyte macrophage cell line HD11 [16] was obtained from Dr. Delphine Beeckman (Ghent University, Belgium) and maintained in Dulbecco’s modified Eagle’s minimal essential medium (DMEM) (Sigma, USA), supplemented with 5% heat-inactivated foetal calf serum, 1% sodium pyruvate, 1% L-glutamine and 0.5% gentamicin (all products from Gibco, USA), at 41°C, 5% CO_2_ and 90% humidity. The cells (10^6^ cells/ml/well) were infected with vvIBDV strain UPM0081 at a multiplicity of infection (MOI) of 0.1 and 0.5 for 3 h at 41°C.

After incubation, the cells were washed, fresh complete medium was added, and further incubated for 3, 21 and 45 h. The uninfected control and the IBDV-infected cells were harvested and subjected to viral load detection using quantitative real-time polymerase chain reaction (qRT-PCR) and an immune-related gene expression study using the multiplex GeXP system.

### Virus inoculation in SPF chickens

Nine-day-old SPF eggs were obtained from the Veterinary Research Institute, Ipoh, Perak, and hatched at the Laboratory of Vaccines and Immunotherapeutics, Institute of Bioscience, Universiti Putra Malaysia (UPM). The trial was conducted in the animal research facility at the Faculty of Veterinary Medicine, UPM. Three-week-old SPF chickens (n = 29) were randomly divided into three groups. The first group (n = 15) was subjected to oculonasal infection with 10^4^ EID_50_ of vvIBDV strain UPM0081, whilst the second group (n = 9) received phosphate-buffered saline (PBS), and were kept as the control. The third group, consisting of five UPM0081-infected chickens, was kept for clinical and mortality observations. Water and feed were provided *ad libitum*. Five birds from the infected group and three birds from the control group were killed and necropsied at 2, 4 and 5 dpi. The spleen and bursae were harvested, observed for lesions and cut into two pieces. One piece was submersed in RNAlater® solution (Ambion, USA) for ribonucleic acid (RNA) isolation and the remaining half was analysed with flow cytometry. The experimental trials were approved by the Animal Care and Use Committee, at the Faculty of Veterinary Medicine, UPM (reference number UPM/IACUC/AUP-R022/2014). Bursae was also fix in 10% formalin, embedded in paraffin, section and stained with Haematoxylin and Eosin (H&E) for histopathological examination. All bursae samples were assigned with the following lesion scoring where lesion scores of 1 representing no lesions; 2 representing mild reduction in overall follicle size, 3 representing moderate reduction in follicle size; 4 representing severe reduction in follicle size; and 5 representing necrosis and follicle atrophy [17].

### Flow cytometry analysis of B cell, T cell, and macrophage populations, in the spleens and bursae of infected and uninfected SPF chickens

The spleens and bursae were harvested at 2, 4 and 5 dpi from both the control group and the infected chickens, and were washed using Hank’s balanced salt solution. Afterwards, the samples were pressed through a 70 μm sterile wire mesh screen (SPL Life Sciences, China). The lymphocytes were isolated using Ficoll-Plaque Plus™ (Amersham Biosciences, USA) according to the recommended protocol by the manufacturer with slight modifications. Purified lymphocytes from each groups were separated into four different tubes and each of them were stained with 10 μg/ml of 10 μl FITC-labelled CD3, PE-labelled CD4, IgM and KUL01 and PerCP-labelled CD8 antibodies (Southern Biotech, USA), respectively, and analysed using a FACSCalibur™ flow cytometer with CellQuest™ Pro software (BD Bioscience, USA).

### RNA extraction and cDNA synthesis

Total RNA from the HD11 cells, spleens and bursae was isolated using an RNeasy® Plus Mini Kit (Qiagen, Germany) according to the protocol provided by the supplier. The quality and quantity of the extracted RNA were determined using NanoDrop™ ND-1000 (Thermo Scientific, Wilmington, USA). SuperScript™ III reverse transcriptase (Invitrogen, USA), and oligo(dT)18 primer (Fermentas, Lithuania) were selected to synthesise the cDNA. Total RNA with the final concentration of 1 μg was used in the cDNA synthesis.

### IBDV quantitative real-time RT-PCR

In order to detect the IBD viral load in infected HD11 cells, spleens and bursae, a forward primer of 5′-ATG CTC CAG ATG GGG TAC TTC-3′ and a reverse primer of 5′-TTG GAC CCG GTG TTC ACG-3′, targeting the VP2 gene of the IBDV, were designed and optimised using iQ™ SYBR® Green Supermix (Biorad, USA). The thermal cycling protocol of polymerase activation and DNA denaturation was 95°C for 3 min, followed by 40 cycles of denaturation of 15 s at 95°C, 30 s of annealing and extension at 58.7°C. The melt curve analysis was 65 to 95°C, at increments of 0.5°C every 5 s/step. No template control (NTC) was included to ensure no cross-contamination during sample preparation. To ensure the specific amplification from IBD viral RNA and not DNA contamination, no-RT control was used. Standard curve was generated in RT-qPCR with the ten-fold serially diluted viral RNA ranged from 9 to 14 log_10_ copies number. The viral load in infected HD11 cells, spleens and bursae was quantify based on the generated standard curve.

### Multiplex GeXP assay

GenomeLab™ GeXP assays were performed to measure 27 selected genes with immune function and glyceraldehyde-3-phosphate dehydrogenase (GAPDH) was used as the reference gene for normalisation according to the method previously reported [18]. Primers to amplify the target genes were designed using the GenomeLab eXpress Profiler software (Table 1), which produced fragment sizes ranging from 137 to 350 nucleotides. The gene expression data were normalised by dividing the peak area of each gene by the peak area of the GAPDH gene and the fold change of expression of each gene was calculated using the following formula: fold change = normalised data of the gene from treated samples/normalised data of the gene from untreated samples [18]. The data for each gene and technical replicate were averaged and calculated.

**Table 1:** **GeXP primers sequence and amplicon sizes designed for quantification of chicken cytokines, chemokines and other immune-related genes**

**Gene**	**Accession number**	**Amplicon size (bp)**	Forward primer sequence* (5′–3′)	Reverse primer sequence** (5′–3′)
***Cytokines and chemokines***				
**CCL4**	NM_204720	235	CTGCTCAAAGCCTGCCATC	GTGCAGCCATCCTGAAGC
**CXCLi1**	NM_205018	228	CCGATGCCAGTGCATAGAG	CCTTGTCCAGAATTGCCTTG
**CXCLi2**	NM_205498	165	CCTGGTTTCAGCTGCTCTGT	GCGTCAGCTTCACATCTTGA
**GM-CSF**	NM_001007078	242	TGAAAACAAATGGGACAGAGG	TTCTCCTCTGGGAGCACATC
**IFN-γ**	NM_205149	214	GAGCCATCACCAAGAAGATGA	TAGGTCCACCGTCAGCTACA
**IL-1β**	NM_204524	137	CCAGAAAGTGAGGCTCAACA	GTAGCCCTTGATGCCCAGT
**IL-2**	NM_204153	144	GTGGCTAACTAATCTGCTGTCCA	CCGTAGGGCTTACAGAAAGG
**IL-4**	NM_001007079	151	CGTCAAGATGAACGTGACAGA	AGGTTCTTGTGGCAGTGCT
**IL-6**	NM_204628	158	AGTTCACCGTGTGCGAGAAC	TTCGTCAGGCATTTCTCCTC
**IL-10**	NM_001004414	172	TAACATCCAACTGCTCAGCTC	TGATGACTGGTGCTGGTCTG
**IL-12α**	NM_213588	179	AAGGGACTCAACTGCTCCAG	TTGTGTTGCTCTGACTGTTGG
**IL-15**	NM_204571	193	ATTCCCGATCCAGATTCTGTT	ACAGTTGGTACTGGAGACAAATACT
**IL-16**	NM_204352	200	GCCTCACAAGAATCAACAACTG	TGCTTTGTTCCAACGAGGTC
**IL-17 F**	NM_204460	340	TCCATGGGATTACAGGATCG	AGGCAAGGCAGTTCTCCTG
**IL-18**	NM_204608	207	CGTCAATAGCCAGTTGCTTG	CTTCTACCTGGACGCTGAATG
**IL-21**	NM_001024835	186	TGTGGTGAAAGATAAGGATGTCG	CAGTTTTGGCGAATGTAGCA
**IL-22**	XM_416079	256	CAGCCCTACATCAGGAATCG	GAACTGTGCCACATCCTCAG
**TGF-β3**	NM_205454	347	AATCAGCATACACTGCCCTTG	TCGGAAGTCAATGTAAAGAGGAC
**TNFSF13B**	NM_204327	333	GGCAAGGTCTCCACTAGAGC	TCAGAAGCCAAGGGACAATG
***Toll-like receptors***				
**TLR2-1**	NM_204278	249	TCAGCTACACCAAAATGTTCAACC	CGTGATTTTGCCTGTGAGC
**TLR3**	NM_001011691	263	TGCATAAGAAGGAGCAGGAAG	CTGGCCAGTTCAAGATGCAG
**TLR4**	NM_001030693	270	CATCTCTGGAGTTCCTGCTG	AGGCTGCTAGACCCAGGTG
**TLR5**	NM_001024586	277	CACTCAGGTTCTCGGTATTCG	AATCCAGGTGCTTCAGCAAG
**TLR7**	NM_001011688	284	GAGTGAGTTATGCCACTCCTCTC	TCAAAGGCTTCCACATCAC
***Others***				
**iNOS**	NM_204961	221	TATGCTCTGCCTGCTGTTGC	ATGCAAGTTTGTTGCTTTCC
**MHCI**	NM_001044683	291	GGAAACCTGCGTGGAGTG	TGGTGACCCAGGTGTGGTA
**MHCII**	NM_001044679	298	AGTACGCGCACTTCGACA	AGAAGCCCGTCACGTAGC
**GAPDH**	NM_204305	312	CTGGCAAAGTCCAAGTGGTG	AGCACCACCCTTCAGATGAG
**KAN** ^r***^	-	325	ATCATCAGCATTGCATTCGATTCCTGTTTG	ATTCCGACTCGTCCAACATC

*Forward universal primer sequence (AGGTGACACTATAGAATA).

**Reverse universal primer sequence (GTACGACTCACTATAGGGA).

***Internal control.

### Quantitative real-time RT-PCR validation of GeXP data

The GeXP data for CXCLi2, IFN-γ, IL-12α and IL-18 gene expression in the spleen and bursae were validated via qRT-PCR, using the primers and probes described by Kaiser *et al.* [19] and Kogut *et al.* [20], with some modification of the PCR reaction protocols (Table 2). The amplification step was carried out in a 25 μl reaction by adding 4 μl of cDNA, 12.5 μl of iQ Supermix (100 mM KCl, 40 mM Tris–HCl, 1.6 mM dNTPs, iTaq DNA polymerase 50 units/ml, 6 mM MgCl_2_, and stabilisers), 1 μl of 10 mM of each primer and 2 μl of 1 mM probe, topped up with sterile distilled water. Amplification and detection of specific products was carried out using CFX96™ Real-Time System (BioRad, USA) with the following cycle profile: 1 cycle at 95°C for 5 min, 40 cycles at 95°C for 20 s, and 58°C or 60°C for 30 s as indicated in Table 2 RNA extracted from HD11 and ConA-C1-Vick T cell lines was used to generate a standard curve by serial dilution (10^-1^ to 10^-5^) [18]. For each qRT-PCR experiment, the test samples and the ten-fold serially diluted RNA were run in duplicate. A no template control was also included. No-template control and no-RT control were also included. Quantification was carried out according to the Pfaffl method with corrected efficiency for each primer set [21].

**Table 2:** **Real-time quantitative RT-PCR probes and primers**

**Target**		**Probe or primer sequence (5′–3′)**	**Accession no.**	**Annealing temperature**
**CXCLi2**	Probe	(FAM)-TCTTTACCAGCGTCCTACCTTGCGACA-(BHQ1)	AJ009800	60°C
	F	GCCCTCCTCCTGGTTTCAG		
	R	TGGCACCGCAGCTCATT		
**GAPDH**	Probe	(FAM^a^)-CGCCATCACTATCTTCCAGG-(BHQ1)	NM_204305	58°C
	F^b^	GAACGGGAAACTTGTGAT		
	R^b^	GACTCCACAACATACTCA		
**IFN-γ**	Probe	(FAM)-TGGCCAAGCTCCCGATGAACGA-(BHQ1)	Y07922	58°C
	F	GTGAAGAAGGTGAAAGATATCATGGA		
	R	GCTTTGCGCTGGATTCTCA		
**IL-1β**	Probe	(FAM)-CCACACTGCAGCTGGAGGAAGCC-(BHQ1)	AJ245728	60°C
	F	GCTCTACATGTCGTGTGTGATGAG		
	R	TGTCGATGTCCCGCATGA		
**IL-10**	Probe	(FAM)-CGACGATTCGGCGCTGTCACC-(BHQ1)	AJ621614	58°C
	F	CATGCTGCTGGGCCTGAA		
	R	CGTCTCCTTGATCTGCTTGATG		
**IL-12α**	Probe	(FAM)-CCAGCGTCCTCTGCTTCTGCACCTT-(BHQ1)	AY262751	58°C
	F	TGGCCGCTGCAAACG		
	R	ACCTCTTCAAGGGTGCACTCA		
**IL-18**	Probe	(FAM)-CCGCGCCTTCAGCAGGGATG-(BHQ1)	AJ276026	60°C
	F	AGGTGAAATCTGGCAGTGGAAT		
	R	ACCTGGACGCTGAATGCAA		

^a^FAM, 6-carboxyfluorescein; BHQ1, Black Hole Quencher.

^b^ *F*, forward primer; *R*, reverse primer.

### Nitric oxide and malondialdehyde assays

The uninfected and vvIBDV-infected spleens and bursae were homogenised, filtered through a 70 μm mesh, and pelleted at 500 xg for 10 min. Supernatants from both the spleen and bursae were subjected to quantification of nitric oxide and malondialdehyde levels. NO was quantified using the Griess assay, where 150 μl of the supernatant was added to 20 μl of Griess reagent (Invitrogen, USA) and 130 μl of deionised water, followed by 30 min incubation at room temperature. The sample’s absorbance was read at 548 nm using a μQuant ELISA Reader (BioTek Instruments, USA). For the malondialdehyde (MDA) determination, 200 μl of supernatant were added to 800 μl of PBS, 25 μl of butylated hydroxytoluene (Sigma, USA), and 500 μl of trichloroacetic acid and 2-thiobarbituric acid (Sigma, USA), followed by 2 h incubation on ice. The mixture was then pelleted at 500 xg for 15 min and 1 ml of the supernatant was collected and added to 75 μl of 0.1 M ethylene diamine tetraacetic acid and 250 μl of 0.05 M 2-thiobarbituric acid (Sigma, USA). Finally, the sample was boiled for 15 min, cooled to room temperature, and the absorbance recorded at 548 nm by the μQuant ELISA Reader. A standard curve for MDA was prepared concurrently using thiobarbituric reactive substances (Sigma, USA).

### Statistical analysis

The results from this study were subjected to one-way ANOVA analysis with Duncan’s post-hoc test using SPSS version 21, where P < 0.05 is considered as significant.

## Results

### In vitro *infection of HD11 cells with vvIBDV*

IBDV in the infected cells was detected by quantitative real-time RT-PCR. As shown in Table 3, IBDV RNA was detected in HD11 cells as early as 6 h post-infection (hpi), with slight increased at 48 hpi.

**Table 3:** **Detection of virus RNA in HD11 cells infected with vvIBDV strain UPM0081 by SYBR Green real-time qRT-PCR**

**Sample**	**Mean Cq**		**Viral copy number (log**_**10**_ **)**	
	**0.1 MOI**	**0.5 MOI**	**0.1 MOI**	**0.5 MOI**
**Uninfected**	ND	ND	ND	ND
**6 hours**	18.89	18.42	14.16	14.29
**24 hours**	18.85	18.28	14.17	14.33
**48 hours**	18.05	17.30	14.40	14.61

Statistical differences between groups were assessed by one-way ANOVA followed by a Duncan post-hoc test.

*ND* = not detected.

### vvIBDV-induced expression of cytokines, chemokines and other immune-related genes by HD11 cells

mRNA expression levels of the pro-inflammatory cytokine IL-1β, the pro-inflammatory chemokines CCL4, CXCLi1 and CXCLi2, and the Th1 cytokines IL-12α and IL-18 were all upregulated in vvIBDV-infected HD11 cells throughout the experiment (Table 4). At both MOI, mRNA expression levels of IL-1β, CCL4, CXCLi2, IL-12α and IL-18 peaked at 24 hpi, whereas those of CXCLi1 peaked at 6 hpi. In contrast, mRNA expression levels of the anti-inflammatory cytokine IL-10 were generally down-regulated.

**Table 4:** **Relative fold changes in gene expression in HD11 cells infected with different MOI of vvIBDV strain UPM0081 at 6, 24 and 48 hpi**

**Gene**	**6 hpi**		**24 hpi**		**48 hpi**	
	**0.1 MOI**	**0.5 MOI**	**0.1 MOI**	**0.5 MOI**	**0.1 MOI**	**0.5 MOI**
***Pro-inflammatory cytokines and chemokines***						
IL-1β	<2	2.33 ± 0.19^a^	5.53 ± 0.37^b^	6.74 ± 1.78^b^	<2	2.92 ± 0.65^a^
CCL4	2.47 ± 1.03^a,b^	4.07 ± 0.64^a,b^	3.89 ± 0.85^b^	5.07 ± 1.17^a^	2.69 ± 0.78^a^	3.29 ± 0.90^a^
CXCLi1	3.39 ± 0.45^a^	4.34 ± 0.54^b^	1.99 ± 0.35^c^	2.32 ± 0.49^c^	<2	<2
CXCLi2	2.24 ± 0.41^a^	2.44 ± 0.27^a^	6.44 ± 0.75^b^	9.02 ± 0.45^c^	3.84 ± 0.40^d^	4.40 ± 1.14^d^
***Th1 cytokines***						
IL-12α	2.10 ± 0.39^a^	3.32 ± 0.78^b^	3.43 ± 0.31^b^	4.32 ± 1.2^b^	<2	<2
IL-18	<2	3.05 ± 0.53^a^	2.46 ± 0.23^b^	3.34 ± 0.50^b^	<2	<2
***Treg cytokine***						
IL-10	-2.44 ± 0.23^a^	-4.17 ± 0.10^b^	-2.00 ± 0.33^c^	-2.86 ± 0.36^d^	<2	-2.22 ± 0.13^a,c^
***Toll-like receptors***						
TLR2-1	<2	<2	<2	<2	<2	<2
TLR3	<2	2.65 ± 0.37^a,b^	2.00 ± 0.90^a^	4.90 ± 0.44^c^	3.22 ± 0.14^b^	5.22 ± 0.45^c^
TLR4	<2	<2	<2	<2	<2	<2
TLR5	<2	<2	<2	<2	<2	<2
TLR7	<2	<2	<2	<2	<2	<2
***Others***						
IL-15	<2	<2	<2	<2	<2	<2
IL-16	<2	<2	<2	-2.13 ± 0.15	<2	<2
iNOS	3.67 ± 0.53^a^	4.73 ± 0.16^b,c^	3.52 ± 1.1^a,b^	7.51 ± 0.73^d^	5.57 ± 0.61^c^	5.68 ± 0.44^c^
MHCI	<2	<2	<2	-2.22 ± 0.22^a^	2.59 ± 1.18^b^	3.13 ± 1.40^b^
MHCII	-2.33 ± 0.08^a^	-3.13 ± 0.08^b^	<2	-4.55 ± 0.14^c^	<2	-5.26 ± 0.09^d^
TGF-β3	<2	<2	<2	<2	<2	<2

Statistical differences between groups were assessed by one-way ANOVA followed by a Duncan post-hoc test. Means labelled with different letters are significantly different, p < 0.05. <2: less than a 2-fold change.

Of the TLRs measured, only the mRNA expression levels of TLR3 were significantly altered post-infection, with levels increasing with time post-infection at both MOI. For MHC class I, mRNA expression levels were upregulated at 48 hpi for both MOI, but downregulated at 24 hpi only for the MOI of 0.5. For MHC class II, at an MOI of 0.1 mRNA expression levels were down-regulated only at 6 hpi, whereas with an MOI of 0.5, mRNA expression levels were increasingly downregulated with time. For iNOS, mRNA expression levels were upregulated at all time-points for both MOIs, with peak expression being at 48 hpi for an MOI of 0.1 and 24 hpi for an MOI of 0.5.

For the other genes measured, there was very little difference in mRNA expression levels, regardless of MOI or time-point.

### qRT-PCR quantification of viral load in infected spleens and bursae

**Figure 1:**
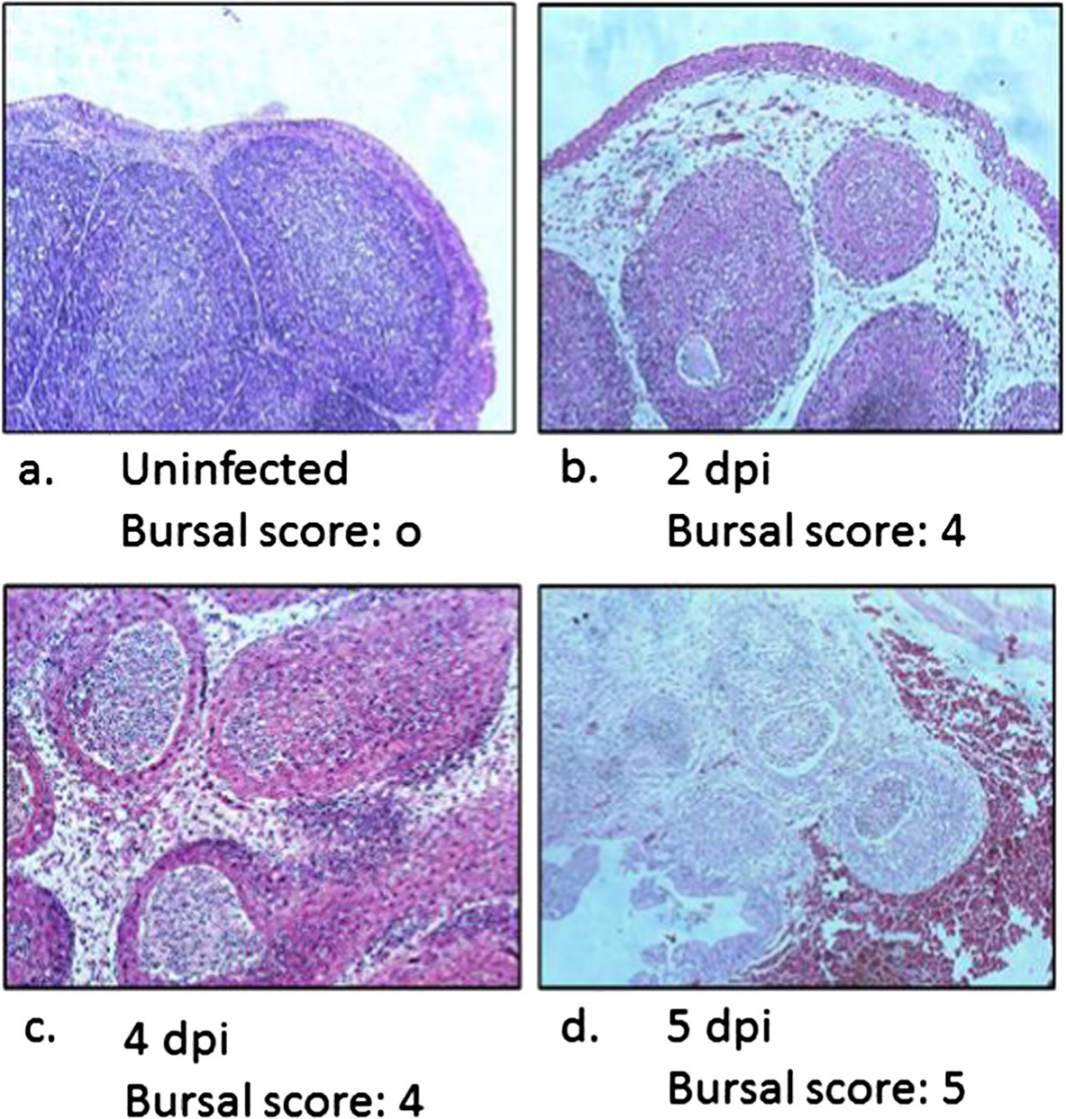
**Bursal histopathology of (a) uninfected, (b) 2 day, (c) 4 day and (d) 5 day post vvIBDV strain UPM0081 infected chicken.** Bursal was scored from 0-5 based on the lesion scoring. (100X).

Clinical signs, including diarrhoea, depression, feather ruffling and loss of appetite, were observed in chickens infected with vvIBDV strain UPM0081, starting from 3 days post-infection (dpi), while 100 percent mortality was observed at 6 dpi. A mottled spleen and bursae haemorrhages were observed in the infected chickens from 3 dpi onwards (Data not shown). Lesion scoring 4 and 5 were recorded in the histology of bursal from vvIBDV strain UPM0081 infected chicken at 4 and 5 dpi, respectively (Figure 1).

Virus in vvIBDV-infected spleens and bursae was detected as early as 2 dpi and peaked at 5 dpi for both spleen and bursae, with 13.49 log_10_ and 13.97 log_10_ copies number, respectively. Viral loads were generally higher in the bursae of Fabricius compared to the spleen, but the differences were not significant (Table 5).

**Table 5:** **Detection of virus RNA in the spleens and bursae of chickens infected with vvIBDV strain UPM0081 by SYBR Green real-time qRT-PCR**

**Sample**	**Mean Cq**		**Viral copy number (log 10)**	
	**Spleen**	**Bursae**	**Spleen**	**Bursae**
**Uninfected**	ND^*^	ND^*^	ND^*^	ND^*^
**Day 2**	30.42 ± 1.78^a^	29.13 ± 2.48^a,b^	10.93^a^	11.29^a,b^
**Day 4**	27.30 ± 0.84^b,c^	25.92 ± 1.60^c^	11.81^b,c^	12.19^c^
**Day 5**	21.29 ± 2.13^d^	19.57 ± 0.21^d^	13.49^d^	13.97^d^

Values are the mean percentages of total cells ± SD per tissue of 5 chickens, each with three technical repeats. Statistical differences between groups were assessed by one-way ANOVA followed by a Duncan post-hoc test. Means labelled with different superscript letters are significantly different (p < 0.05) between organs at different sampling days.

^*^ *ND* = not detected.

### Flow cytometry immunophenotyping of vvIBDV-infected spleens and bursae

In order to study changes in the percentage of T and B lymphocytes and macrophages isolated from the bursae and spleens, the cells harvested from bursae or spleen were counted and stained with antibodies against CD3^+^/CD4^+^, CD3^+^/CD8^+^, IgM^+^ and KUL01^+^ and analysed by flow cytometry. Overall, IBDV infection caused a significant increase in the percentage of KUL01^+^ cells (macrophages), CD3^+^/CD4^+^ T cells and CD3^+^/CD8^+^ T cells in the bursae of Fabricius (Table 6).

**Table 6:** **The cell number of IgM** ^**+**^ **cells, KUL01** ^**+**^ **macrophages, CD3** ^**+**^ **CD4** ^**+**^ **cells and CD3** ^**+**^ **CD8** ^**+**^ **cells in bursae and spleens of 3-week-old chickens infected with vvIBDV strain UPM0081 at 2, 4 and 5 dpi**

		**Bursae (x10** ^**6**^ **cell/bursae)**	**Spleen (x10** ^**6**^ **cell/spleen)**
Average number of cells	Day 0	230.00 ± 2.73^a^	190.00 ± 3.41^a^
	Day 2	173.00 ± 3.64^b^	150.00 ± 2.76^b^
	Day 4	116.00 ± 1.66^c^	66.40 ± 2.31^c^
	Day 5	54.00 ± 1.37^d^	34.40 ± 2.32^d^
IgM^+^	Day 0	189.83 ± 0.83^a^	57.36 ± 1.21^a^
	Day 2	138.99 ± 0.50^b^	44.35 ± 0.15^b^
	Day 4	72.21 ± 0.55^c^	23.27 ± 1.00^c^
	Day 5	35.52 ± 3.07^d^	14.57 ± 1.55^d^
KUL-1^+^	Day 0	9.90 ± 0.52^a^	6.26 ± 0.13^a^
	Day 2	8.76 ± 0.22^a^	18.54 ± 0.93^b^
	Day 4	20.68 ± 2.16^b^	2.08 ± 0.31^c^
	Day 5	12.92 ± 3.50^c^	2.75 ± 0.49^c^
CD3^+^CD4^+^	Day 0	1.73 ± 0.12^a^	41.49 ± 0.80^a^
	Day 2	1.67 ± 0.09^a^	33.04 ± 1.51^b^
	Day 4	3.82 ± 0.43^b^	16.52 ± 2.12^c^
	Day 5	4.25 ± 0.48^b^	7.58 ± 0.53^d^
CD3^+^CD8^+^	Day 0	1.67 ± 0.14^a^	62.36 ± 0.61^a^
	Day 2	1.86 ± 0.30^a^	48.68 ± 0.98^b^
	Day 4	3.05 ± 0.38^b^	20.87 ± 0.66^c^
	Day 5	3.52 ± 0.20^b^	6.81 ± 0.38^d^

The number of cell (mean ± SD) of the cell subsets were determined by trypan blue cell count and flow cytometry using a FACSCalibur with CellQuest Pro software (BD Bioscience, USA). Statistical differences between groups were assessed by one-way ANOVA followed by a Duncan post-hoc test. Means labelled with different superscript letters are significantly different (p < 0.05) between organs at different sampling days.

In the bursae of chickens infected with vvIBDV strain UPM0081, the KUL01^+^ macrophage population increased from ~9.90x10^6^ cells in the control birds to 20.68x10^6^ and 12.92x10^6^ cells at 4 and 5 dpi, respectively (Table 6). However, there was no indication of an increased macrophage population at 2 dpi in the infected bursae. In contrast, IBDV induced an increased in the KUL01^+^ macrophage population in the spleen from 6.26x10^6^ cells in the control to 18.54 x10^6^ cells at 2 dpi. However, the splenic macrophage cell number in infected chickens decreased sharply at 4 and 5 dpi (Table 6).

An increased in CD3^+^/CD4^+^ and CD3^+^/CD8^+^ T cells was also detected in the infected bursae, reaching peaks of 4.25x10^6^ and 3.52x10^6^ cells, respectively, at 5 dpi (Table 6). However, the population of splenic CD4^+^ and CD8^+^ cells were initially high and reduced significantly at 5 dpi (Table 6). IgM^+^ cells in the bursae of infected chickens reduced significantly from 189.83x10^6^ cells in the control to 35.52x10^6^ cells at 5 dpi. Similarly, the splenic IgM^+^ cell number decreased from 57.36x10^6^ cells in control birds, to 14.57x10^6^ cells at 5 dpi (Table 6).

### Expression profiles of immune-related genes in IBDV-infected spleens and bursae

A multiplex quantitative GeXP assay was used to quantify the mRNA expression levels of immune-related genes in the spleens and bursae of SPF chickens infected with vvIBDV strain UPM0081 at 2, 4 and 5 dpi. Of the 27 genes evaluated in this study, 20 genes were significantly regulated (>2-fold up or down) in the bursae and spleen post-infection (Table 7). In general, IBDV infection induced significant upregulation of pro-inflammatory cytokines and chemokines and Th1 cytokines in both tissues.

**Table 7:** **Relative fold changes in gene expression in bursae and spleens of SPF chickens infected with vvIBDV strain UPM0081 at 2, 4 and 5 dpi, using a multiplex quantitative GeXP assay**

	**2 dpi**		**4 dpi**		**5dpi**	
	**Bursae**	**Spleen**	**Bursae**	**Spleen**	**Bursae**	**Spleen**
***Pro-inflammatory cytokines and chemokines***						
IL-1β	<2	7.17 ± 2.17^b^	<2	3.26 ± 1.18^a^	<2	9.31 ± 2.59^b^
IL-6	2.22 ± 0.37^a^	6.51 ± 0.60^b,c^	10.96 ± 3.52^d^	3.35 ± 0.76^a,b^	9.74 ± 3.48^c,d^	2.28 ± 0.40^a^
CCL4	<2	2.61 ± 0.67^a,b^	4.93 ± 2.05^b,c^	2.29 ± 0.30^a^	5.69 ± 2.53^c^	2.19 ± 0.78^a^
CXCLi1	<2	2.16 ± 0.39^a^	4.24 ± 2.24^b^	2.45 ± 0.62^a,b^	2.23 ± 0.44^a^	2.59 ± 0.51^a,b^
CXCLi2	3.56 ± 1.54^a^	2.84 ± 0.95^a^	10.34 ± 3.64^b^	2.86 ± 1.20^a^	4.45 ± 1.95^a^	2.70 ± 1.18^a^
***Th1 cytokines***						
IFN-γ	9.55 ± 2.29^a^	12.30 ± 4.75^a^	26.20 ± 5.23^b^	9.29 ± 4.24^a^	26.22 ± 6.78^b^	9.87 ± 2.92^a^
IL-12α	4.76 ± 2.69^a^	23.95 ± 7.96^b^	10.63 ± 2.58^a^	15.52 ± 9.23^a,b^	11.62 ± 3.25^a^	8.06 ± 4.53^a^
IL-18	5.69 ± 1.92^a^	11.16 ± 2.04^b^	4.03 ± 1.03^a^	4.38 ± 1.59^a^	4.90 ± 3.01^a^	3.97 ± 1.35^a^
***T cell proliferative cytokines***						
IL-2	<2	<2	-3.57 ± 0.09	<2	<2	<2
IL-15	<2	<2	<2	<2	<2	2.39 ± 0.59
***Treg cytokines***						
IL-10	<2	<2	<2	<2	2.10 ± 0.13	<2
***Toll-like receptors***						
TLR3	<2	<2	-2.33 ± 0.18	<2	<2	<2
TLR7	-2.00 ± 0.11^a^	<2	-3.70 ± 0.09^b^	<2	<2	<2
***Others***						
iNOS	2.32 ± 0.50^a^	2.53 ± 0.52^a^	4.27 ± 0.70^b,c^	2.87 ± 0.91^a,b^	4.65 ± 1.29^c^	3.81 ± 0.72^a,b,c^
MHCI	<2	<2	2.14 ± 0.30^a^	2.24 ± 0.35^a^	<2	<2
MHCII	<2	<2	2.93 ± 1.01^a^	2.08 ± 0.49^a^	<2	<2
TGF-β3	<2	-2.00 ± 0.13^a^	-5.26 ± 0.07^b^	<2	-2.44 ± 0.10^a^	<2
TNFSF13B	<2	<2	2.83 ± 0.82^a^	2.46 ± 0.62^a^	3.30 ± 1.50^a^	3.11 ± 0.43^a^
IL-16	<2	<2	-2.04 ± 0.21^a^	-2.56 ± 0.10^a^	<2	<2
IL-17 F	<2	<2	<2	<2	<2	2.00 ± 0.22

Statistical differences between groups were assessed by one-way ANOVA followed by a Duncan post-hoc test. Means labelled with different superscript letters are significantly different (p < 0.05) between organs at different sampling days <2: less than a 2-fold change.

In the bursae of infected birds, the mRNA expression levels of the pro-inflammatory cytokine IL-1β were not statistically different from those in controls. All of the other pro-inflammatory cytokines and chemokines have increased mRNA expression levels, at least at some time-points post-infection, but with different patterns. IL-6 and CXCLi2 mRNA expression levels peaked at 4 dpi and were upregulated throughout the experiment. CCL4 and CXCLi1 mRNA expression levels were only upregulated from 4 dpi, but peaked at 5 and 4 dpi, respectively. Again, mRNA expression levels of the Th1 cytokines measured were upregulated compared to controls at all time-points post-infection. IL-18 was consistently upregulated, whereas IFN-γ and IL-12α mRNA expression levels increased until 4 dpi but then plateaued. mRNA expression levels of the T cell proliferative cytokines IL-2 and IL-15, and the anti-inflammatory (Treg) cytokine IL-10 were essentially unaltered at most time-points from levels in the controls.

Expression levels of mRNA for the two TLRs measured were either downregulated (at 2 and 4 dpi for TLR7 and 4 dpi for TLR3) or unaltered compared to levels in controls. MHC class I and II mRNA expression levels were only upregulated at 4 dpi, and unaltered at the other time-points. iNOS mRNA levels showed a similar pattern of expression to those of IFN-γ and IL-12α.

Expression levels of the other molecules measured were varied. TGF-β3 mRNA expression levels were downregulated at 4 and 5 dpi, whereas those of the TNF superfamily member 13B were upregulated at the same time-points. IL-16 mRNA expression levels were slightly downregulated at 4 dpi, whereas those of IL-17F were unaltered compared to those in controls.

The spleen showed strong upregulation of the mRNA expression levels of pro-inflammatory cytokines and chemokines, Th1 cytokines and iNOS at all time-points, but there are few other changes compared to levels in controls. Upregulation of IL-1β mRNA expression levels appeared to be biphasic, higher at 2 and 5 dpi than at 4 dpi. IL-6 mRNA expression levels peaked at 2 dpi. The mRNA expression levels of the pro-inflammatory chemokines are consistently upregulated throughout the experiment but do not alter with time. Expression levels of mRNA for the three Th1 cytokines also peaked at 2 dpi. iNOS mRNA expression levels increased throughout the course of the experiment and peaked at 5 dpi.

Expression levels of the T cell proliferative and anti-inflammatory cytokines measured were largely unaltered in the spleen, as were levels of the TLRs measured, TGF-β3 and IL-17F. mRNA expression levels for MHC class I, MHC class II and TNFSF13B were all upregulated at 4 dpi (and at 5 dpi also for the latter), whereas IL-16 mRNA expression levels were downregulated at 4 dpi.

### Validation of the GeXP gene expression profiles using qRT-PCR

**Figure 2:**
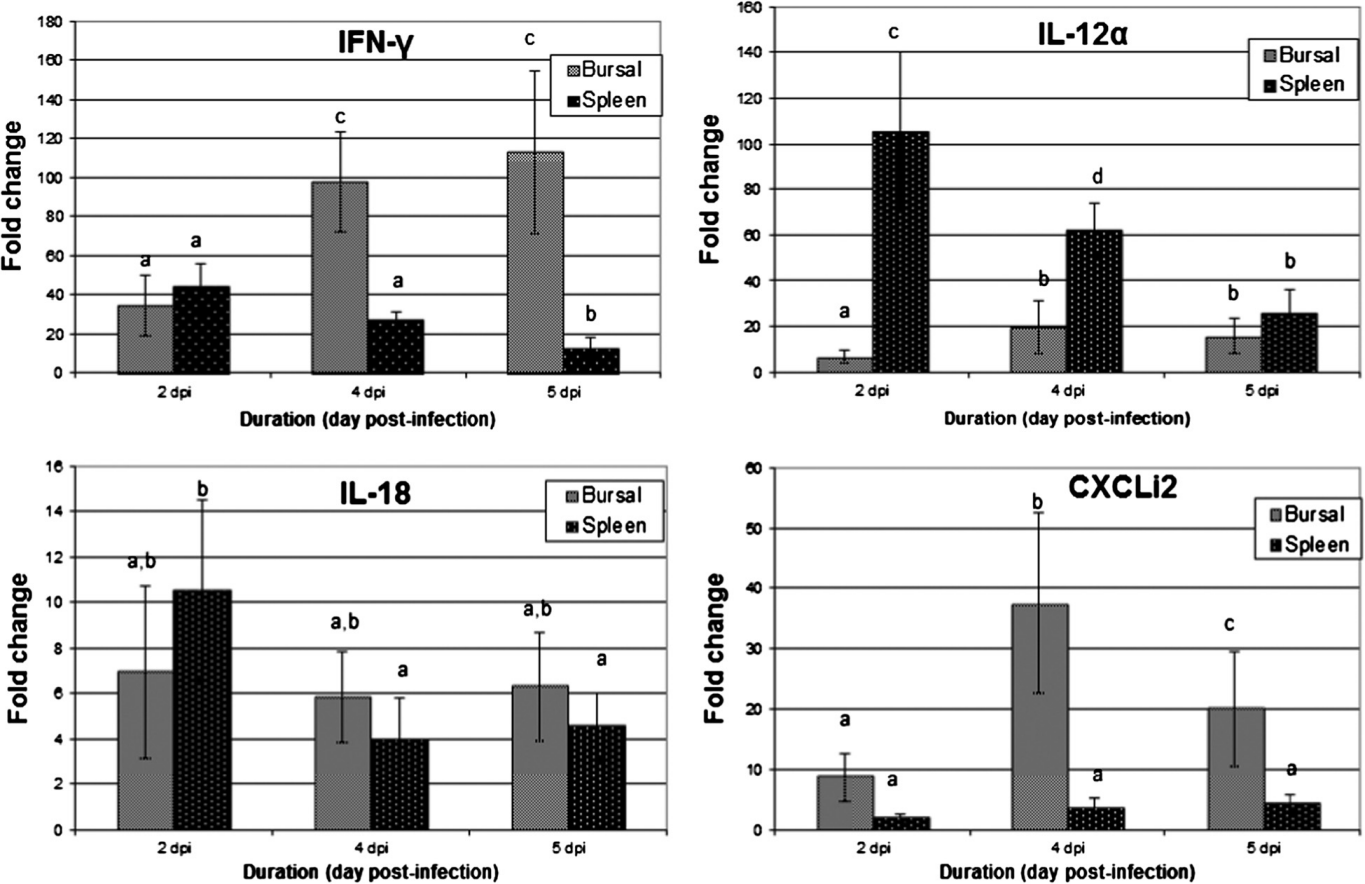
**Differential mRNA expression levels of IL-12α, IL-18, CXCLi2 and IFN-γ, as determined by qRT-PCR, in the bursae of Fabricius of SPF chickens infected with vvIBDV strain UPM0081 at 2, 4 and 5 dpi.** Results are represented as fold change compared to levels in uninfected controls, after normalization with GAPDH (glyceraldehyde-3-phosphate-dehydrogenase) calculated by the − ΔΔCq method [20]. Groups labelled with different letters are significantly different, p < 0.05.

In order to confirm the data obtained from the GeXP multiplex assay, qRT-PCR was performed to quantify the mRNA expression levels of selected cytokines, namely IFN-γ, IL-12α, IL-18 and CXCLi2 (Figure 2). Overall, qRT-PCR detected higher fold changes than the GeXP assay in all of the genes quantified, but confirmed the expression patterns previously detected by the GeXP assay (Table 7).

In the bursae of chickens infected with vvIBDV strain UPM0081, the Th1 cytokines IFN-γ and IL-12α were upregulated as early as 2 dpi (Figure 2). The highest upregulation of IFN-γ mRNA levels was 113-fold at 5 dpi (Figure 2). IL-18 mRNA expression levels were upregulated from 2 dpi and remained unchanged until 5 dpi. CXCLi2 mRNA expression levels were up-regulated 9-fold as early as 2 dpi, peaked at 4 dpi at 40-fold, but decreased to 20-fold at 5 dpi (Figure 2).

In the spleens of chickens infected with vvIBDV strain UPM0081, IFN-γ mRNA expression levels were up-regulated 44-fold at 2 dpi and decreased thereafter (Figure 2). IL-12α mRNA expression levels were 106-fold up-regulated at 2 dpi, but decreased thereafter to 26-fold at 5 dpi. IL-18 mRNA expression levels were up-regulated moderately after infection, with a peak of 11-fold at 2 dpi. CXCLi2 mRNA expression levels were up-regulated at all time-points (up to 4-fold at 5 dpi).

### NO and MDA levels in vvIBDV-infected spleen and bursae

**Figure 3:**
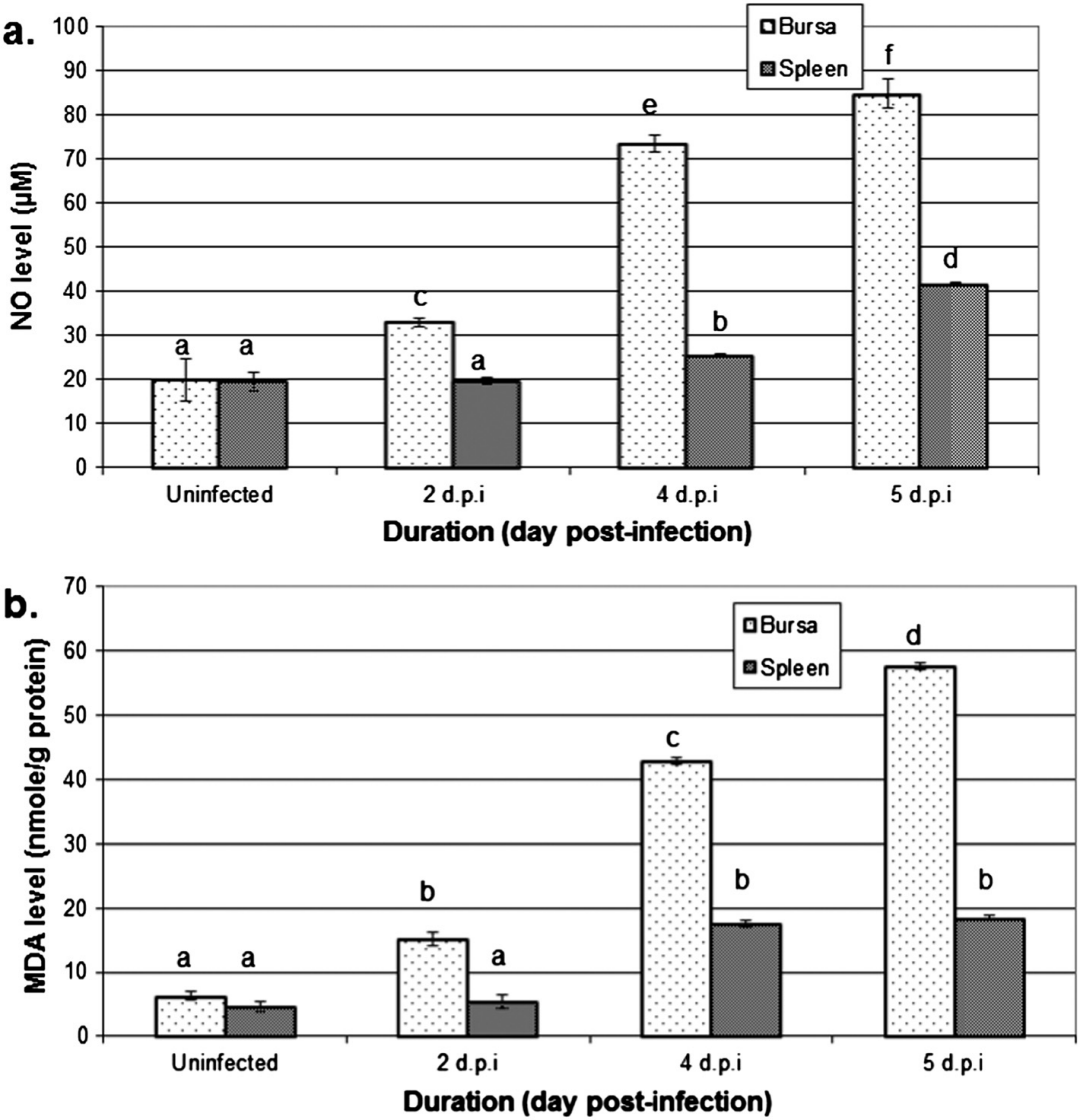
**Level of inflammatory mediator (a) nitric oxide (NO) and lipid peroxidation reactive aldehydes (b) malondialdehyde (MDA) in the spleens and bursae of chickens infected with vvIBDV strain UPM0081 at 2, 4 and 5 dpi.** Differences between control and treated groups were determined by one-way ANOVA (p < 0.05). Means labelled with different letters are significantly different, p < 0.05.

Nitric oxide, which acts as a pro-inflammatory mediator and the production of which is often used as a measure of IFN-γ activity, was up-regulated in both the bursae and spleens of vvIBDV-infected chickens (Figure 3a). NO levels in the bursae increased more than those in the spleen and in both organs peaked at 5 dpi. Similarly, the lipid peroxidation rate in vvIBDV-infected spleens and bursae was measured using malondialdehyde as an oxidative stress marker. Similarly to NO, at 3 dpi MDA levels in the bursae increased throughout the experiment (Figure 3b), whilst levels in the spleen were also elevated but only at 4 and 5 dpi, with there being no significant difference between levels at those latter two time-points (Figure 3b).

## Discussion

IBDV infection is associated with up-regulation of pro-inflammatory cytokines and destruction of actively dividing IgM^+^ B lymphocytes [8,10]. These previous studies looked at alterations of expression of a limited panel of cytokines, chemokines and TLRs following infection with classical and very virulent strains of IBDV [8,10]; we believe this is the most exhaustive study to date, and the first to compare responses to *in vitro* infection of macrophage-like cells (HD11 cell line) with those to *in vivo* infection in the spleen and bursae. Changes in the numbers of macrophages, T and B cells in the two tissues following infection were also quantified.

It has been proposed that macrophages play a critical role in spreading IBDV from the gut to the bursae [22]. This study illustrates that vvIBDV infection of HD11 cells can be detected as early as 6 h after adding virus to the cells, and that viral load was maximal at the end of the experiment, 48 hpi. After *in vivo* infection with vvIBDV, virus was detected in both the spleen and bursae as early as 2 dpi using qRT-PCR, confirming an earlier study that detected the IBDV genome in the spleen and bursae as early as 3 dpi [10]. Increased viral load in both the spleen and bursae was associated with the progression of the disease and histological changes in the respective tissues (Figure 1). Although the load of IBDV was higher in the bursae than in the spleens, these results were not statistically significant (Table 5), indicating both spleen and bursae are equally susceptible to vvIBDV infection. This study also confirms a previous study by Rautenschlein *et al.* [23] that showed that infection with virulent strains of IBDV are associated with severe lesions in non-bursae organs, including the spleen.

IBDV infection is associated with activation of innate and adaptive antiviral immune responses, via proliferation of different effector cells, including macrophages and T cells [10]. Although similar viral loads were observed in the vvIBDV-infected spleens and bursae, kinetics of changes in macrophage numbers were different in the two organs (Table 6). In the infected spleens, there was a rapid increase in macrophage numbers at 2 dpi, followed by a marked reduction at 4 dpi back to levels in control spleens. In the infected bursae, macrophage numbers were increased at 4 and 5 dpi. This finding supports an earlier study, where bursae macrophage numbers increased significantly at 3 and 5 dpi [24]. The decreased in macrophage numbers in the spleen at 4 dpi could reflect migration of splenic macrophages to the bursae. Khatri and Sharma [25] reported that acute infection by IBDV was associated with lysis of macrophages that contributed to a reduction in innate immunity, followed by a rapid recovery of macrophage numbers and a revival of innate immunity. Changes in macrophage numbers in the infected tissues could consequently alter the expression levels of various cytokines and chemokines, which in turn could enhance the infiltration of heterophils, macrophages [25] and T cells [12] into the bursae. In this study, increasing numbers of CD4^+^ and CD8^+^ T cells, as similarly reported by Kim *et al.* [12], were also observed in the bursae from 4 to 5 dpi. The CD4^+^ T and CD8^+^ T cells may have migrated from the spleen, as there was a significant decrease in numbers of these cells in the spleen at 5 dpi (Table 6). However, as has been previously suggested [26], it is still not clear whether the changes in cell numbers in the vvIBDV-infected bursae were the result of cell migration to/from other tissues or the periphery, or if there were expansions/contractions of the resident cell populations.

Expression of immune genes in response to *in vitro* infection of HD11 cells was remarkably similar to expression of immune genes in the bursae and spleen following *in vivo* infection, although the precise kinetics of expression sometimes differed slightly for the same gene between the different cells and tissues. In HD11 cells, spleens and bursae, infection with vvIBDV strain UPM0081 induced increased mRNA expression levels, in a dose-dependent manner, of pro-inflammatory cytokines and chemokines, Th1 cytokines, iNOS and MHC class I. This result is similar as previously reported [24,25] where IBDV infection promoted upregulation of proinflammatory cytokines including IL-1β, IL-6, IL-18 and iNOS of adherent putative macrophage cell isolated from bursae of IBDV challenged chicken. This is typical of the immune response to infection with a virus – an innate immune response is triggered, followed by a Th1 response leading to increased viral antigen presentation in the context of MHC class I, and the production of effector molecules, such as NO through the induction of iNOS.

Differences from this pattern were slight but the main ones will be discussed. In the *in vitro* HD11 cell infection model, mRNA expression levels of TLR3, which recognises dsRNA (IBDV is a dsRNA virus), were also upregulated. MHC class II mRNA expression levels were down-regulated, as were those of the anti-inflammatory cytokine IL-10, indicating that the macrophage-like cells were switched to a strong Th1-promoting anti-viral phenotype for the HD11 *in vitro* model. The tissues studied in the *in vivo* infection model are of course a complex mix of immune and non-immune cells. In the bursae, in contrast to infected HD11 cells, mRNA expression levels of both TLR3 (recognises double-stranded viral RNA) and TLR7 (recognises single-stranded viral RNA) were downregulated. Based on previous report by Rauf et al. [10], TLR3 reacted differently post-infected with different strains of IBDV where upregulation of TLR3 was recorded post-classical IBDV infection but in contrary downregulation was observed post-variant IBDV infection. Another study also indicated that response of TLR3 may play an important role in resistance of the indigenous chicken against vvIBDV infection [27]. mRNA expression levels of both MHC class I and II were upregulated, but only transiently, whereas IL-10 mRNA expression levels were unaltered from those in controls. In the spleens, mRNA expression levels of the TLRs were unaltered from those in controls, but levels of both MHC class I and II were upregulated. IBDV has been reported to inhibit immunoproliferation of spleen CD4 and CD8 T cell via non MHC-restricted interaction [28]. Furthermore, upregulation may delay the recovery of IBDV infected chicken since MHC-II was found to restrict T-cell dependent secondary antibody response against IBDV [29]. Different pattern of TLR 3 and IL10 responses maybe contributed by the complex interaction of immune and non-immune cell in both spleen and bursae. Although HD11 has been widely used as the chicken macrophages cell line model for cytokines study and has shown similar pattern in proinflammatory response post LPS stimulation, the nature of HD11 as a cell line transformed by avian myelocytomatosis virus (MC29) [16,30] may contribute to the slight differences in gene expression as noticed in this study.

Parallels can be drawn between this study and previous studies measuring the immune response to IBDV infection. In this study, IL-15 mRNA expression levels were unaltered, as previously reported [8]. Levels of expression of the mRNA for TLR3 and TLR7 were downregulated at 3 dpi, again as previously described [10]. IL-16 is a chemoattractant for CD4^+^ T cells, and is also involved in the development of B cells in the bursae [31]. IBDV infection is associated with down-regulation of IL-16 [27]. In this study, IL-16 was down-regulated at 4 dpi in infected spleens and bursae. Upregulation of IFN-γ expression in infected bursae and spleens was detected throughout the experiment, again consistent with previous studies [23,32], and as stated above suggesting the involvement of inflammatory and cell-mediated responses in the immune response to infection and possibly the associated tissue destruction/immunopathology seen in the bursae following IBDV infection. T cells infiltrate IBDV-infected bursae tissue to clear the virus [13]. However, this infiltration and induction of local inflammation may exacerbate bursae lesions and thus delay bursae recovery [11]. Previous report has stated the important of TLR3 in controlling the virus replication through coordinating stimulation of proinflammatory mediators (including proinflammatory cytokines and type II interferon) and antiviral mediators (such as type I interferon). Inhibition of TLR3-mediated antiviral response may subsequently lead to non-TLR3-mediated recognition that resulting in increased proinflammatory responses associated with increased virus replication [33] which is similar to the results in this study.

The substantial increase in the mRNA expression levels of pro-inflammatory cytokines presumably drove the increased iNOS mRNA expression levels and increased the levels of NO, which subsequently contributed to oxidative stress, as indicated by the levels of lipid peroxidation in the infected organs (Figure 3). As reported by Akaike and Maeda [34], oxidative stress and NO induce immune cell apoptosis. Therefore, our results suggest that oxidative stress may also play an important role in the impairment of the bursae and lymphocyte function during IBDV infection.

Understanding on the role of cytokines to the pathogenesis of vvIBDV offers the potential to design better vaccine against the prevention of disease. For example, this study has reported the importance of TLR3 in IBD progression similar to other previous findings [10,27]. Thus, it is possible to design a TLR3 ligands as a potential adjuvant to the current vaccine since previous study has reported the success of using TLR3 ligand poly-ICLC to enhance vaccine efficacy in mouse model [35]. Besides, enhancing other T cell proliferating cytokines such as IL-2, which was found downregulated, via preparing plasmid DNA vaccine adjuvant may help to improve the efficacy of vaccine to stimulate better cell-mediated immunity against vvIBDV infection [36].

## Conclusion

In summary, this study indicated that cells of the macrophage-like cell line, HD11, infected with vvIBDV provide a good model to study the overall effects of *in vivo* vvIBDV infection, at least as occur in the bursae and spleen, suggesting that macrophages may play an important role in promoting inflammation and tissue damage in the vvIBDV-infected chicken. Future studies should elucidate the precise identities and roles of the cells producing the differentially expressed immune molecules described in this study and the relative contribution of macrophages in immunopathology of vvIBDV infection.
